# Association Between Prescription Opioid Use and Mortality in Community-Dwelling Older Adults

**DOI:** 10.1093/geroni/igaa057.666

**Published:** 2020-12-16

**Authors:** Carina D’Aiuto, Helen-Maria Vasiliadis

**Affiliations:** 1 Université de Sherbrooke, Longueuil, Quebec, Canada; 2 University of Sherbrooke, Sherbrooke, Quebec, Canada

## Abstract

Prescription opioid use is concerning among older adults. Yet, few studies have examined the impact of opioid use on mortality by considering multimorbidity. Our sample includes 1586 older adults aged ≥65 recruited in primary care from 2011-2013 in a large health administrative region in Quebec and participating in the ESA-Services study, a longitudinal study on aging and health service use. An opioid prescription delivered in the 3 years prior to the baseline interview was identified using the provincial pharmaceutical drug registry. Mortality was ascertained from the vital statistics registry until 2015. The presence of chronic diseases was based on self-reported and physician diagnostic codes in health administrative databases. Physical multimorbidity was defined as ≥3 chronic physical conditions from either source. Physical/psychiatric multimorbidity was defined as ≥3 chronic physical conditions and ≥1 common mental disorder from either source. Logistic regression analyses were conducted to examine the association between opioid use and mortality, controlling for sociodemographic factors. Interactions were tested for opioid use and multimorbidity. Older adults with physical multimorbidity using opioids were 1.76 (95%CI: 1.02-3.03) times more likely to die than those not using opioids. Those with physical/psychiatric multimorbidity using opioids were 2.27 (95%CI: 1.26-4.09) times more likely to die than those not using opioids. Older age, male sex, and single marital status significantly increased the risk of mortality. Overall, opioid use increases the risk of death in older adults with multimorbidity. The presence of mental disorders further increases the risk of death in older adults with physical multimorbidity using opioids.

